# The burden of alcohol-related morbidity and mortality in Ottawa, Canada

**DOI:** 10.1371/journal.pone.0185457

**Published:** 2017-09-28

**Authors:** Jacqueline Willmore, Terry-Lynne Marko, Darcie Taing, Hugues Sampasa-Kanyinga

**Affiliations:** Ottawa Public Health, Ottawa, Ontario, Canada; National Institue on Drug Abuse, UNITED STATES

## Abstract

**Objectives:**

Alcohol-related morbidity and mortality are significant public health issues. The purpose of this study was to describe the prevalence and trends over time of alcohol consumption and alcohol-related morbidity and mortality; and public attitudes of alcohol use impacts on families and the community in Ottawa, Canada.

**Methods:**

Prevalence (2013–2014) and trends (2000–2001 to 2013–2014) of alcohol use were obtained from the Canadian Community Health Survey. Data on paramedic responses (2015), emergency department (ED) visits (2013–2015), hospitalizations (2013–2015) and deaths (2007–2011) were used to quantify the acute and chronic health effects of alcohol in Ottawa. Qualitative data were obtained from the “Have Your Say” alcohol survey, an online survey of public attitudes on alcohol conducted in 2016.

**Results:**

In 2013–2014, an estimated 595,300 (83%) Ottawa adults 19 years and older drank alcohol, 42% reported binge drinking in the past year. Heavy drinking increased from 15% in 2000–2001 to 20% in 2013–2014. In 2015, the Ottawa Paramedic Service responded to 2,060 calls directly attributable to alcohol. Between 2013 and 2015, there were an average of 6,100 ED visits and 1,270 hospitalizations per year due to alcohol. Annually, alcohol use results in at least 140 deaths in Ottawa. Men have higher rates of alcohol-attributable paramedic responses, ED visits, hospitalizations and deaths than women, and young adults have higher rates of alcohol-attributable paramedic responses. Qualitative data of public attitudes indicate that alcohol misuse has greater repercussions not only on those who drink, but also on the family and community.

**Conclusions:**

Results highlight the need for healthy public policy intended to encourage a culture of drinking in moderation in Ottawa to support lower risk alcohol use, particularly among men and young adults.

## Introduction

Alcohol-related morbidity and mortality are significant public health issues worldwide [1]. The estimated worldwide consumption in 2010 was equal to 6.2 litres of pure alcohol consumed per person aged 15 years or older, which translates into 13.5 grams of pure alcohol per day. In 2012, about 3.3 million deaths– 5.9% of all global deaths—were attributable to alcohol consumption [2]. In 2013, an estimated 22 million Canadians, almost 80% of the population, reported that they drank alcohol in the previous year [3]. The total cost of alcohol-related harm to Canadians has been estimated to be $14.6 billion per year, of which alcohol-related health care costs alone total $3.3 billion [4]. Alcohol use is associated with a wide range of acute and chronic health and social consequences [1, 5]. More specifically, alcohol consumption has been related to greater risk of injuries, violence, suicide, poisoning, cirrhosis, certain cancers and strokes [6]. The risk of alcohol-related harms varies by drinking patterns, which have been shown to differ by several sociodemographic and socioeconomic (SES) characteristics, such as age, sex, income and education [2, 7, 8]. For example, men are well known to be more likely to consume alcohol and consume it more often and in larger quantities than women [9–11]. As a result, they have greater negative health outcomes including morbidity, mortality, and disability than women [12]. In addition, research has shown that although individuals with higher education and income drink more alcohol than those with low income, it is people of lower education and income who are more at risk of alcohol-related harms [13–15].

Despite these well-established health and social risks related to alcohol consumption, the Ontario provincial government responsible for the majority of alcohol regulation has been expanding access and availability of alcohol since 2014, on the basis of promoting local industry, convenience and personal choice for consumers [16]. A municipal alcohol policy (MAP) provides policy direction for the sale, service and consumption of alcohol on City property or at locations or events under the City’s control. The City of Ottawa updated their MAP in November of 2016 and recommended residents follow Canada's Low-Risk Alcohol Drinking Guidelines (LRADG). The LRADG recommend no more than two drinks a day, 10 per week for women, and three drinks a day, 15 per week for men, with an extra drink allowed on special occasions. The LRADG help Canadians moderate their alcohol consumption and reduce their immediate and long-term alcohol-related harm.

Research has shown that increasing the availability of alcohol is related to increased consumption and alcohol-attributable morbidity and mortality [17–20]. Further, alcohol-related harms have repercussions that can affect others in the community [21]. A previous public intercept survey of adults conducted in 2015 in public venues in Ottawa found that drunk driving, violence, underage drinking and binge drinking were commonly identified concerns related to the impact of alcohol use in the community. Although alcohol is the most commonly misused substance in Ottawa [22], information on the spectrum of alcohol consumption, associated health harms and community impact is necessary to better inform future intervention and prevention efforts.

The objectives of this study were (1) to provide an overview of alcohol consumption in Ottawa, Ontario, Canada, (2) to describe the prevalence and trends over time of alcohol-related morbidity and mortality in Ottawa, Ontario, Canada and (3) to provide an overview of local perspectives and impacts of alcohol use on families and the community.

## Methods

### Alcohol consumption and attributable morbidity and mortality

Prevalence (2013–2014) and trends (2000–2001 to 2013–2014) of alcohol use data were obtained for the sample of Ottawa (N = 1,724) and Ontario-less-Ottawa (N = 33,827) from the Canadian Community Health Survey (CCHS), a repeated cross-sectional national telephone survey. People living on Indian Reserves or Crown Lands, living in prisons or health care facilities, full-time members of the Canadian Forces, or living in certain remote areas were excluded. A multistage sampling strategy was employed and approximately 98% of the target population was sampled. A complete description of the methods of the survey is published elsewhere [23]. The following sociodemographic variables were collected and were considered for analysis of alcohol consumption: age, sex, mother tongue, language, income and immigration status. Analysis focussed on adults ages 19 and older because this is the legal drinking age in Ontario [24].

Binge drinking is defined as consuming five or more drinks on a single occasion for males and four or more for females. Heavy drinking is defined as binge drinking at least once a month in the past year.

Alcohol-related risks were categorized as previously described by Thomas (2012): No risk = no alcohol use in past year; Low risk = no binge drinking in past year; Moderate risk = binge drinking three times or less a month in past year, and; High risk = binge drinking weekly or more often in past year [25].

Data on paramedic responses (2015), emergency department (ED) visits (2013–2015), hospitalizations (2013–2015) and deaths (2007–2011) were used to quantify the acute and chronic health effects of alcohol on individuals in Ottawa. Data on paramedic responses to alcohol ingestion or intoxication were obtained from the Ambulance Dispatch Reporting System by Ottawa Paramedic Service. A point density map of paramedic responses was created for alcohol-related calls per square kilometre in Ottawa. Emergency department visits were extracted from the National Ambulatory Care Reporting System (Canadian Institute for Health Information). Hospitalization data were derived from the Discharge Abstract Database (Canadian Institute for Health Information) and the Ontario Mental Health Reporting System. Mortality data were from the Vital Statistics database (Office of the Registrar General).

Alcohol-attributable ED visits is a measure that describes illness and injuries that were completely (100%) attributable to alcohol, such as alcohol poisoning or alcohol use disorders. The alcohol-related diagnosis the physician makes at the end of the ED visit can be divided into four categories: (1) mental health conditions (e.g. alcohol use disorder, alcohol withdrawal), (2) chronic disease (e.g. alcoholic liver disease, alcoholic gastritis), (3) alcohol poisoning (i.e. intoxication), and (4) fetal alcohol spectrum disorder. Categories 1, 2, and 4 are chronic effects, while category 3 is considered acute effects [26].

Alcohol-attributable hospitalization (AAH) is a measure that describes serious illness attributed to alcohol. Alcohol consumption is considered a contributing factor for all injuries included and some injuries were completely (100%) attributable to alcohol (e.g., alcohol poisoning). Some neuropsychiatric (referred to as mental health) and chronic conditions such as alcoholic psychosis, alcohol dependence and alcoholic gastritis are 100% attributable to alcohol.

For most chronic conditions, however, alcohol is a contributory factor and measures of the fraction of cases attributable to alcohol were used to estimate the full impact of alcohol on the health of people in Ottawa. Similar to the AAH, alcohol-attributable mortality (AAM) describes how many deaths (rather than hospitalizations) occur in a population that can be attributed to alcohol use. For 100% attributable conditions, all ages were used; however, for partially attributable conditions, only those aged 15 to 69 years could be included due to methodological limitations [4, 27]. International Classification of Disease (ICD-10) codes were used to classify alcohol-attributable ED visits, hospitalizations and deaths (S1 Table) [28]. For partially attributable conditions, the attributable fractions were calculated following the methodology described by Rehm et al [27].

### Public attitudes towards alcohol

Using an interpretive approach to better understand more about alcohol concerns in Ottawa, OPH conducted a mixed methods online survey of local attitudes on alcohol in 2016, called the “Have Your Say” survey (N = 1832). The survey was advertised through social media, partner organizations and earned media and utilized convenience sampling. Prior to data collection, the survey received approval from Ottawa Public Health’s Research Ethics Committee. Individuals aged 16 years or older who lived, worked or attended school in Ottawa were eligible to complete the survey between February 1 and March 14, 2016 via FluidSurveys (SurveyMonkey, San Mateo, California). Participation was voluntary and respondents consented to participate prior to initiating the survey. Respondents were informed that stories might be used by OPH later. To ensure anonymity, all identifiable information was removed from collected quotations. The questionnaire included demographic information such as age, language and the first three digits of postal code.

Four close-ended questions were asked to validate the level of concern from the top four concerns—drunk driving, violence, binge drinking and underage drinking—identified in the 2015 public intercept survey. The following response options were provided; no concern, somewhat concerned, concerned, strongly concerned, not sure. An open-ended follow-up question was asked after each of these four questions for the participant to explain why the item was of concern. Respondents were asked another open-ended question “*As a community*, *what do you think is the top alcohol concern we should work on and why*?*”* A definition of culture of drinking in moderation was provided as “*Those who choose to drink understand how much*, *when and when not to drink*, *will drink for the right reasons in the right place*, *know what the risks are and how to lower them and do not harm themselves*, *their families or their community”* and respondents were asked “*Do you think Ottawa has a culture of moderation*?” and if “*yes”* respondents were asked “*Why*?*”* in an open-ended text field. Finally, respondents were asked “*Have you or others been affected by alcohol use in our community*? *Tell us how*.*”*

### Data process and analysis

Univariate analyses of CCHS alcohol consumption data were weighted and accounted for the complex survey design within STATA (version 14.0, Stata Corp., College Station, Texas). The level of statistical significance was set at *P*<0.05. Qualitative data were analyzed using NVivo 9 version 9.2.81.0 and thematic analysis by a single researcher to consistently code and theme findings.

## Results

### Alcohol consumption and attributable morbidity and mortality

Table 1 presents the percentage of Ottawa adults aged 19 years and older who reported alcohol consumption in the past year, binge drinking, exceeding weekly limits, and heavy drinking by sociodemographic characteristics. An estimated 595,000 adults in Ottawa or 83% of the adult population drank alcohol in the past year, significantly higher than the rest of Ontario (78%). Forty-two percent of adults reported binge drinking in the past year and 20% reported binge drinking at least once every month (i.e. heavy drinking). About 22% of adults reported exceeding the recommended weekly LRADG alcohol limits. Men were more likely than women to report alcohol consumption in the past year, binge drinking, heavy drinking, and to exceed weekly limits. Young adults (aged 19–24 years) were more likely to report binge drinking and heavy drinking compared to other ages. Respondents in the highest income group were more likely to report alcohol consumption in the past year, engage in binge drinking and to exceed weekly limits. Those with a mother tongue other than English or French were less likely to engage in binge drinking, heavy drinking and to exceed the weekly limits. Immigrants were less likely than non-immigrants to report alcohol consumption in the past year, binge drinking, heavy drinking, and to exceed weekly LRADG limits.

**Table 1:** Percentage of adults (19 years and older) who reported alcohol use in the past year, binge drinking, exceeding weekly limits, and heavy drinking by sociodemographic characteristics, Ottawa, 2013–2014.

	Alcohol consumption in the past year		Binge drinking		Heavy drinking		Exceeding weekly limits	
	%	p value	%	p value	%	p value	%	p value
Region		0.002		0.077		0.215		0.264
Ottawa **(N = 1,724)**	82.7		42.2		19.5		22.3	
Ontario less Ottawa **(N = 33,827)**	77.5		38.9		17.6		20.5	
Sex		0.002		0.003		0.036		0.001
Men	87.6		47.7		23.2		27.1	
Women	78.2		37.1		16.1		17.9	
Age		0.158		<0.001		<0.001		0.262
19–24	88.2		67.0		44.1		30.1	
25–44	83.1		53.7		21.5		21.9	
45–64	83.4		34.4		16.0		20.6	
65+	76.6		16.2		5.3*		21.7	
Income		<0.001		0.002		0.053		0.001
Lowest	61.3		38.2*		NR		NR	
Lower Middle	78.2		37.8*		21.4*		12.3*	
Upper Middle	69.7		26.6		10.5*		13.9	
Highest	88.9		47.6		21.8		26.6	
Mother tongue		<0.001		<0.001		<0.001		<0.001
English	89.2		50.7		24.3		28.2	
French	89.6		50.5		24.7		23.7	
Other	65.6		20.5		7.5*		10.2*	
Immigration status		<0.001		<0.001		0.002		<0.001
Non-immigrant	89.1		51.2		24.4		27.3	
Immigrant	68.9		23.5		10.1		11.6*	

*Interpret with caution—high sampling variability. Vertical bars represent 95% confidence intervals.

**Data source:** Canadian Community Health Survey 2013 to 2014, Statistics Canada, Share File, Ontario Ministry of Health and Long-Term Care.

**Fig 1:**
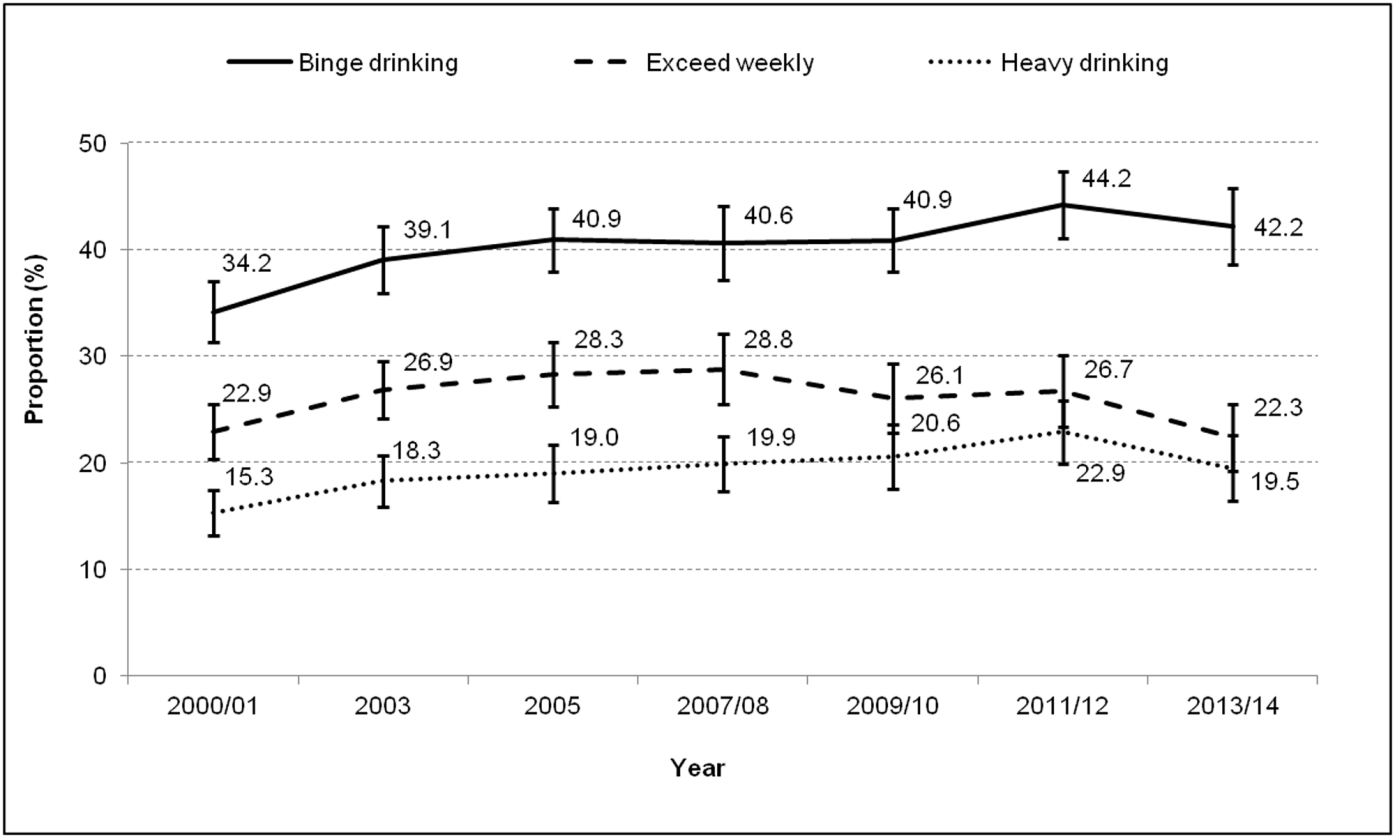
Percentage of adults (19 years and older) who reported binge drinking, heavy drinking, or exceeding weekly limits of Canada’s LRADG, by year, Ottawa, 2000–2014.

The percentage of adults who reported binge drinking, heavy drinking, or exceeding weekly limits of Canada’s LRADG over time (from 2000 to 2014) are displayed in Fig 1. Overall, the prevalence of binge drinking increased from 34% in 2000–2001 to 42% in 2013–2014. Heavy drinking increased from 15% in 2000–2001 to 20% in 2013–2014. The proportion of adults that exceeds weekly limits decreased from 29% in 2007–2008 to 22% in 2013–2014.

**Fig 2:**
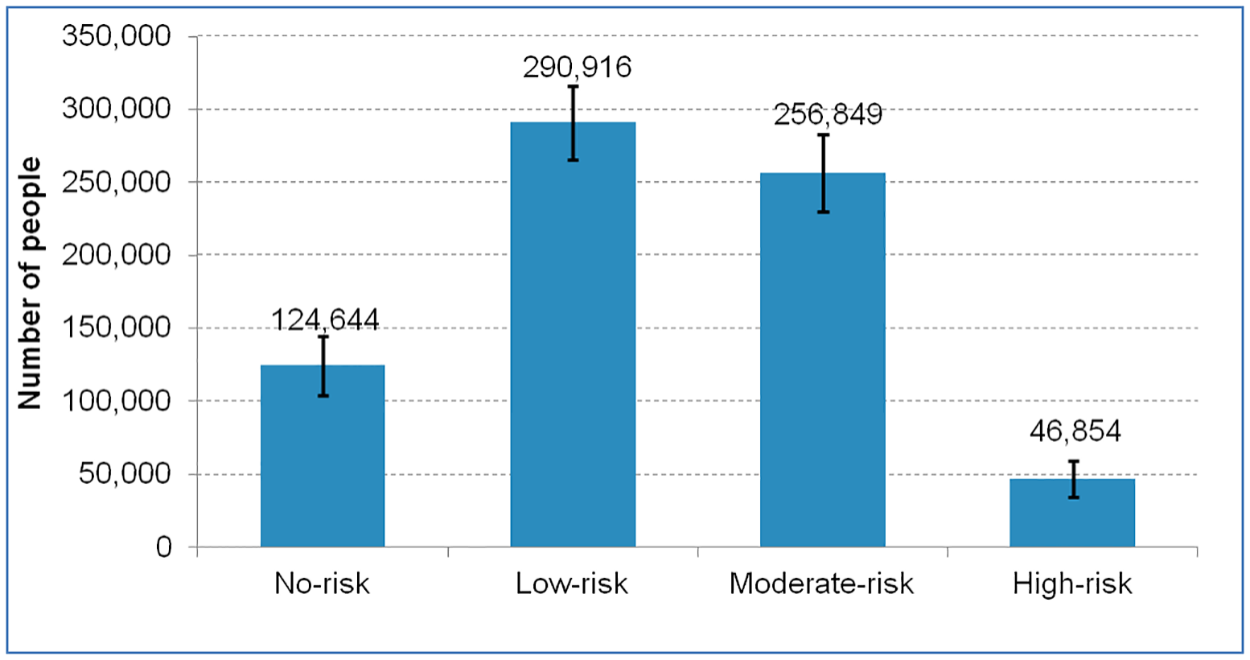
Distribution of alcohol-related risk among adults (19 years and older), Ottawa, 2013–2014. **Note:** No risk = no alcohol use in past year; Low risk = no binge drinking in past year; Moderate risk = binge drinking three times or less a month in past year, and; High risk = binge drinking weekly or more often in past year.

Fig 2 presents the distribution of alcohol-related risk among adults aged 19 years and older in Ottawa in 2013–2014. Approximately 304,000 adults in Ottawa (42% of adults) were considered to be at moderate to high risk of alcohol-related harm. There were 5.5 times as many moderate risk drinkers as high-risk drinkers in Ottawa. Nearly 291,000 of adults (40.4%) were at low risk.

**Fig 3:**
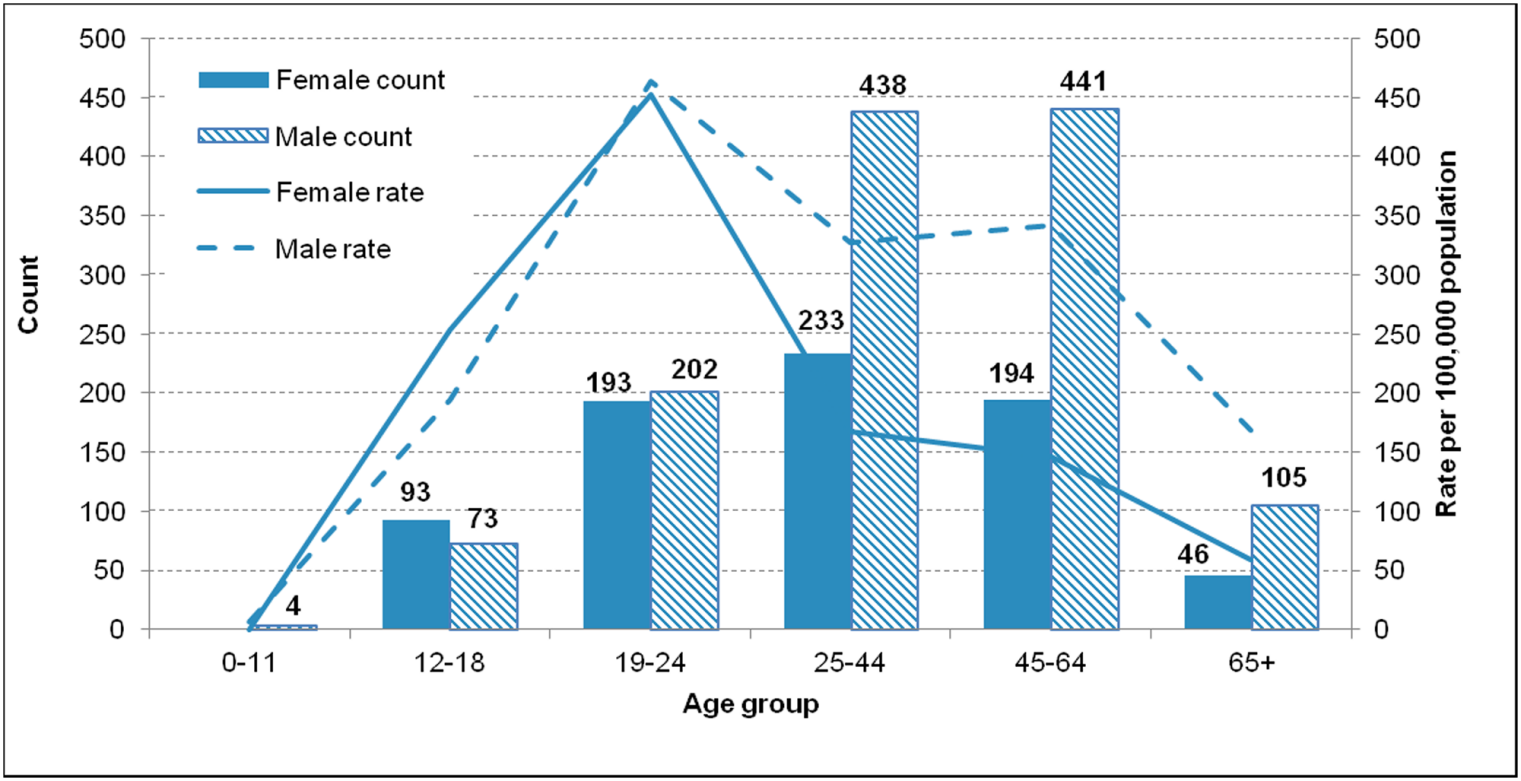
Counts and rates of alcohol-related paramedic responses by age group and sex, Ottawa, 2015.

**Fig 4:**
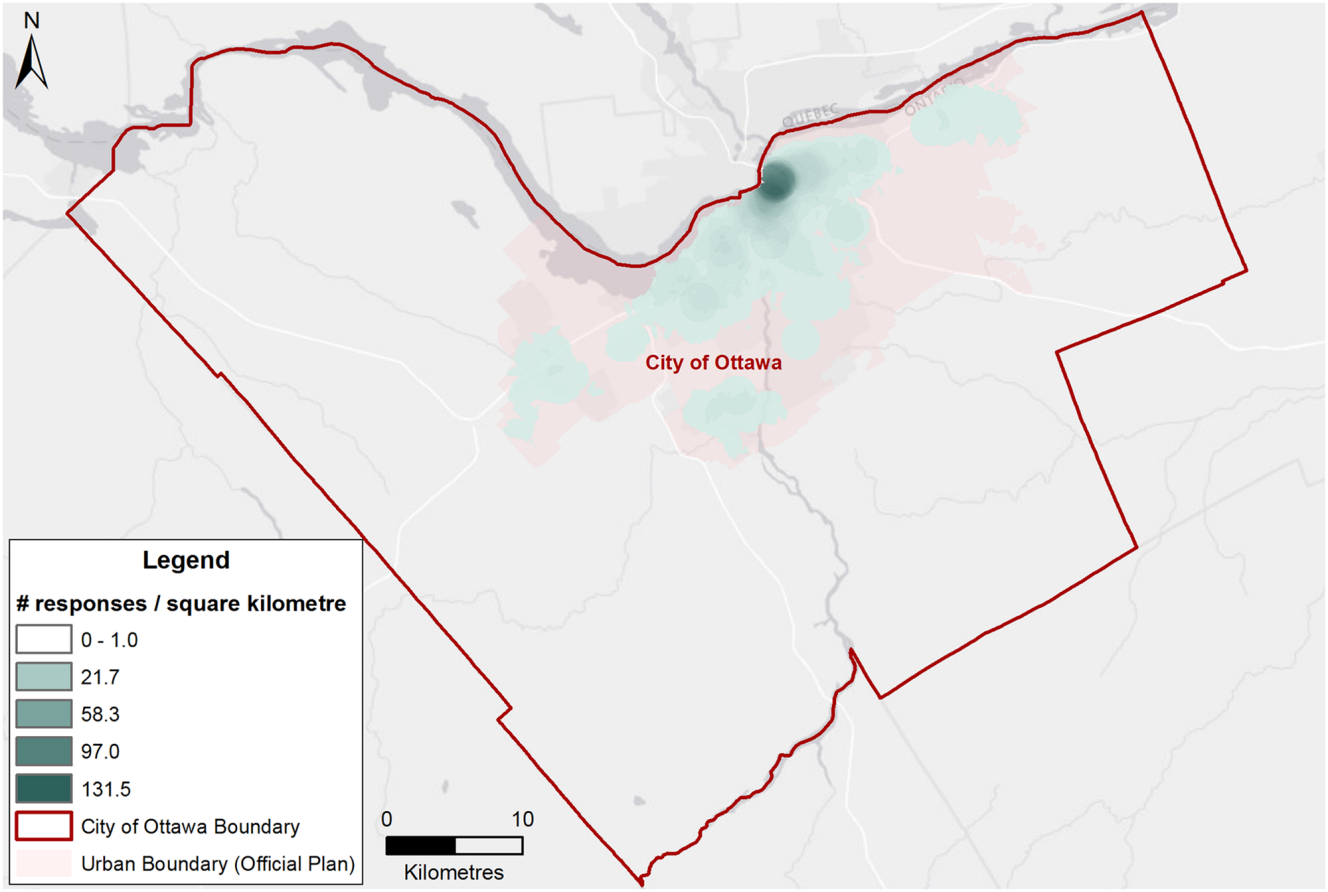
Point density ward map of paramedic responses for alcohol-related calls per square kilometre, Ottawa, 2015. **Note:** Darker shading indicates a higher number of alcohol-related incidents per square kilometre.

In 2015, the Ottawa Paramedic Service responded to 2,060 calls directly attributable to alcohol. Men accounted for a higher number and rate of alcohol-related paramedic responses compared to females, particularly among those aged 25 and older (Fig 3). Young adults aged 19 to 24 years represented the highest rate of alcohol-related paramedic responses—this age group had the highest rates for both men (463.9 per 100,000) and women (452.5 per 100,000). However, men aged 25 to 64 years contributed the highest number of calls (n = 879). Paramedic responses were concentrated in the downtown core of Ottawa, but with some responses in most wards (Fig 4). The highest density of responses for 2015 was 131.5 responses per square kilometre.

Table 2 presents the annual average number of alcohol-attributable ED visits (2013–2015), hospitalizations (2013–2015), and deaths (2007–2011) by diagnosis and sex. On average, there were approximately 6,100 ED visits per year due to alcohol. Men have higher rates of alcohol-attributable ED visits than women. Mental health conditions were the leading cause of ED visits due to alcohol. Annually, there were an average of 1,270 hospitalizations in Ottawa and 140 deaths. Men account for 63% and 70% of the alcohol-related hospitalizations and deaths, respectively. Injuries (e.g. alcohol-related falls) and mental health conditions such as alcohol psychoses and alcohol dependence were the leading cause of alcohol-related hospitalization at 36% (451 hospitalizations) and 35% (440 hospitalizations) of alcohol-related hospitalizations, respectively. Injuries (e.g. alcohol-related suicide, motor vehicle collisions, or falls) were the leading cause of alcohol-related death at 40% (56 deaths).

**Table 2:** Annual number of alcohol-attributable ED visits (2013–2015), hospitalizations (2013–2015), and deaths (2007–2011) average by diagnosis and sex, Ottawa.

	Total		Men		Women	
	Count	%	Count	%	Count	%
**ED visits**	**6081**	**100.0**	**3931**	**100.0**	**2149**	**100.0**
Mental health conditions	5674	93.3	3677	93.5	1997	92.9
Chronic disease	388	6.4	263	6.7	124	5.8
Alcohol poisoning	107	1.8	53	1.4	54	2.5
Fetal alcohol syndrome	10	0.2	6	0.1	4	0.2
**Hospitalizations**	**1269**	**100.0**	**801**	**100.0**	**470**	**100.0**
Injuries	451	35.6	278	34.7	173	36.8
Mental health conditions	440	34.6	284	35.4	156	33.1
Digestive diseases	183	14.4	124	15.5	59	12.5
Cancer	70	5.5	37	4.6	35	7.4
Low birth weight or FASD	66	5.2	33	4.1	33	7.1
Cardiovascular disease	59	4.6	45	5.6	14	3.0
Other chronic disease	1	0.1	1	0.1	0	0.1
**Death**	**139**	**100.0**	**98**	**100.0**	**42**	**100.0**
Injuries	56	40.4	40	40.3	17	39.5
Digestive diseases	39	28.3	29	29.2	11	25.4
Cancer	22	15.6	14	14.8	9	20.1
Mental health conditions	17	11.9	11	11.5	5	12.5
Cardiovascular disease	5	3.7	4	4.3	1	2.4

ED: emergency department; FASD—fetal alcohol spectrum disorder.

*Note*—numbers may not add up to total due to rounding.

**Fig 5:**
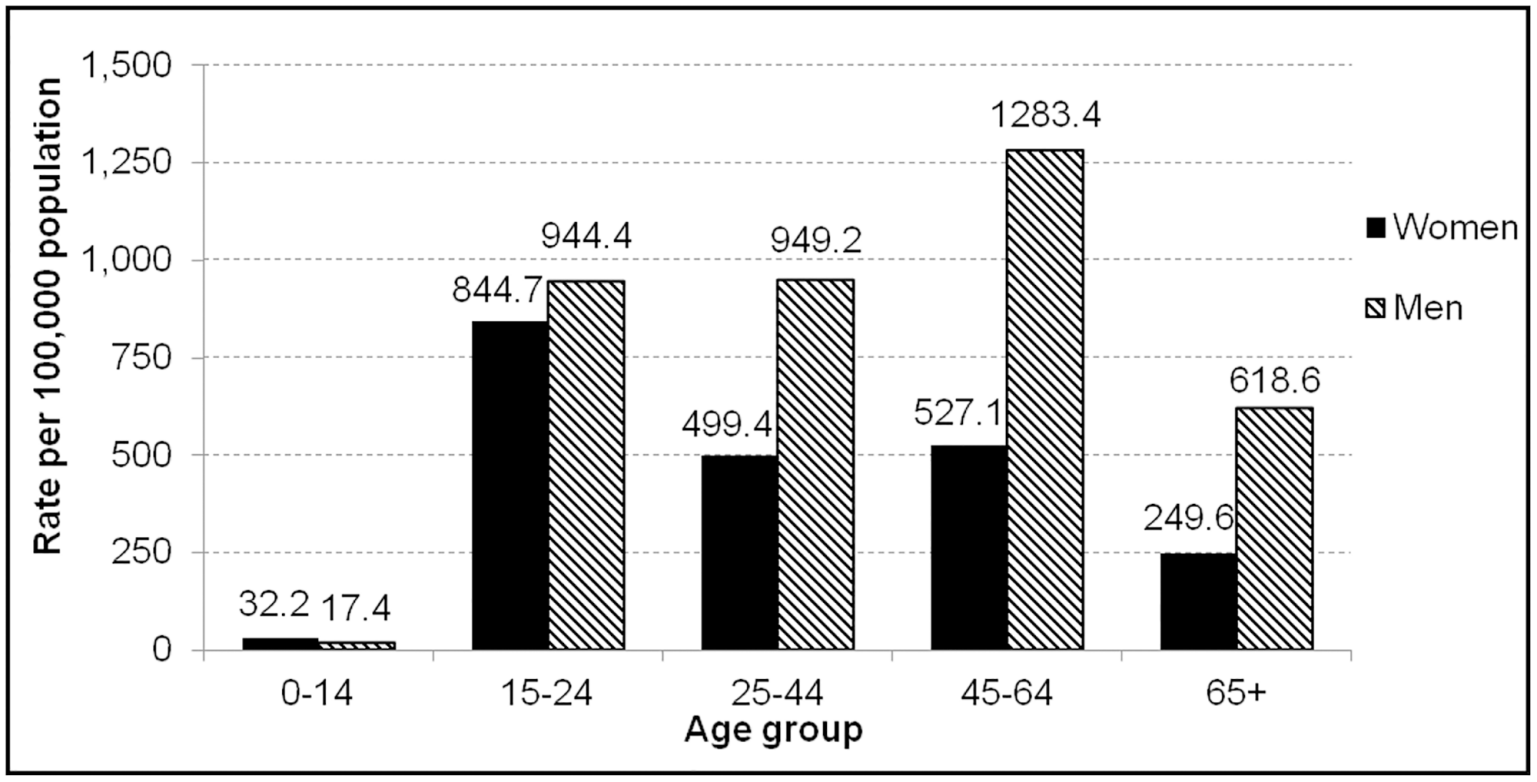
Age-specific rate of 100% alcohol-attributable ED visits by sex, 2013–2015, Ottawa.

Fig 5 presents age-specific rate of 100% alcohol-attributable ED visits (2013–2015) by sex and age. Overall, men had 1.8 times as many ED visits due to alcohol than women (3,931 per year vs. 2,149 per year). The highest rate overall is seen among men aged 45 to 64 years (1283.4 per 100,000), but among women the highest rate is among those aged 15 to 24 years (844.7 per 100,000).

### Public attitudes towards alcohol

A total of 1832 people 16 years and older responded to at least one question of the “Have Your Say” alcohol survey. Respondents in each age category included; less than 25 years (29%), 25–44(34%), and 45 years and older (37%). The majority of respondents completed the survey in English (80%), and were from central Ottawa (39%). The levels of concern with drunk driving, violence, binge drinking, and underage drinking are summarized in Table 3. Overall, 90%, 76%, 56%, and 48% of respondents were concerned or strongly concerned about drunk driving, alcohol-related violence, binge drinking, and underage drinking, respectively. Nineteen per cent of respondents identified no concern with underage drinking and 15% had no concern for binge drinking.

**Table 3:** Levels of concerns with drunk driving, violence, binge drinking, and underage drinking in Ottawa, 2016.

	No concern		Somewhat concerned		Concerned		Strongly concerned		Not sure	
	Count	%	Count	%	Count	%	Count	%	Count	%
Binge drinking (N = 1391)	211	15.2	382	27.4	346	24.9	432	31.0	21	1.5
Violence (N = 1388)	94	6.8	224	16.1	350	25.2	711	51.2	9	0.6
Drunk driving (N = 1392)	35	2.5	96	6.9	209	15.0	1043	75.0	8	0.6
Underage drinking (N = 1388)	268	19.3	431	31.1	295	21.3	375	27.0	19	1.4

#### Drunk driving

When asked “Why do you feel that way?” about drunk driving, 66% (915 of 1391) of respondents shared a written response. The majority of respondents were concerned with drunk driving because of its affect on others: the themes of “innocent people”, “families”, “other drivers”, and the “system” emerged.

*“Drunk driving affects a lot of people*. *I have lost friends due to drunk driving and I believe there is a need to have a greater punishment as well as educational programs.”*

(Respondent aged between 16 to 18 years)

*“Killing innocent people and maybe themselves*. *My daughter lost her fiancé on Christmas Eve to a drunk driver… A life time of grief.”*

(Respondent aged 45 years and older)

A theme of “negative attitude” also arose as written responses about drunk driving were seen as “preventable”, “unacceptable” and “happening too often”.

*“Huge concern as drunk driving is selfish, irresponsible, and preventable*. *If a person wants to drink that's fine, however when they decide to place someone else in harm’s way by driving, that is concerning and unacceptable.”*

(Respondent aged between 19 to 24 years)

#### Alcohol-related violence

When asked “Why do you feel that way?” about violence and alcohol, 63% (880 of 1388) of respondents provided a written response. Common themes that arose concerning alcohol and violence included “concern for others”, specifically for “the innocent”, “safety of the public”, and “personal safety”.

*“This is very concerning to a community because it means the streets aren’t safe*. *We have to protect the vulnerable of the community such as children.”*

(Respondent aged between 16 to 18 years)

Written responses about alcohol-related domestic violence also arose as a theme, where violence was happening behind closed doors or not reported.

*“Unfortunately, I think most drinking related violence stays at home—people are being abused behind closed doors by violent alcoholics and little can be done to help them if they aren't willing to help themselves”*.

(Respondent aged between 25 to 44 years)

Alcohol was indicated to be an important correlate of violence. Specifically, themes of reasons why included alcohol “changes behaviours”, “reduces inhibitions”, “inevitably leads to violence”, and “makes violent people more violent”. Bars and public events were identified as locations where alcohol-related violence may be more frequent or where prevention efforts could occur.

*“I've noticed in Ottawa (since I have called 911 before) that after people are let out of the bars, they tend to get very violent for no apparent reason*. *They take it out on others in the area and even hurt their friends/ bfs/ gfs. It usually starts with verbal violence, then escalates into physical depending on the situation.”*

(Respondent aged between 19 to 24 years)

#### Binge drinking

When asked “Why do you feel that way?” about binge drinking, 66% (913 of 1392) of respondents provided a written response. The most common themes that emerged as to why respondents were concerned about binge drinking was “risk to the individual”; specifically, “concerns for individual health”, “vulnerability to violence (including sexual violence)”, and “future addiction”. Themes of “risks to others” and “on the system” were less common.

*“*“*binge drinking is an indicator for underlying traumas and lack of ability to express emotions and participate as part of a community fabric*, *in my opinion*. *In societys where people feel repressed or aren't emotionally expressive*, *we see this a lot*. *Also depression*, *loneliness*, *and lack [o]f community cohesion*. *So I see this as indicating a need for culture and lifestyle shifts that can be facilitated by community infrastructure and programming*, *though of course aren't entirely due to that*.*”*

(Respondent aged 25 to 44 years)

Of the 15% (211 of 1392) respondents who had no concern with binge drinking, 39% (83 of 211) were aged less than 25 years. One hundred and sixty-seven written responses as to why they had no concern included the themes “it is not an issue” or “not an issue that affects me” as most frequent responses.

“[binge drinking] It's not any worse than any other type of consumption.”

(Respondent aged between 16 to 18 years)

*“I don't see binge drinking as an issue because in our community it does not seem to be a pressing issue*. *It is someone's choice to drink as much as they want. Its their responsibility to know their limits.”*

(Respondent aged between 19 to 24 years)

#### Underage drinking

When asked “Why do you feel that way?” about underage drinking, 64% (894 of 1388) provided written responses. Common themes of why underage drinking is a concern included its “link to other negative behaviours or harms”, including “violence”, “addiction” as well as “negative health effects”.

*“Poisoning, dangerous behaviour due to impaired thinking in an already vulnerable population can have horrific, long term consequences*. *start rewarding good behaviour instead of old school and ineffective punishment”*

(Respondent aged 45 years and older)

A theme of “negative attitude” emerged about underage drinking as indicated by terms in many responses such as “unacceptable”, “too common”, “easily accessible to youth”, and “related to overdrinking”. Themes of “parental acceptance” and “peer pressure” arose as influencers on underage drinking.

In contrast, 19% (268 of 1388) of respondents identified no concern with underage drinking. Of those, 51% (138 of 268) were aged less than 25 years with the theme of “it’s not an issue” that emerged most frequently.

*“I don’t have much concerns about underage drinking because I feel its not really hurting anyone*. *I don’t think one year or two will make that much of a difference in how responsible and safe a person is.”*

(Respondent aged between 16 to 18 years)

#### Culture of moderation

When asked to respond with yes, no or unsure to the question “Do you think Ottawa has a culture of moderation?” 869 provided a response. Less than a third of the respondents (259 of 869) stated “Yes” Ottawa has a culture of alcohol moderation. Of those who did not feel Ottawa has a culture of moderation (320 of 869), 279 provided a written response why. The most frequent themed reasons included; “frequency of alcohol-related harms”, including “drunk driving”, “violence”, “addiction”, “fetal alcohol spectrum disorder”, “frequency of binge drinking”, “association between alcohol use and all social activities”, and “high prevalence of alcohol use in post secondary students and underage youth”.

#### Alcohol-related harms and concerns to work on first

**Fig 6:**
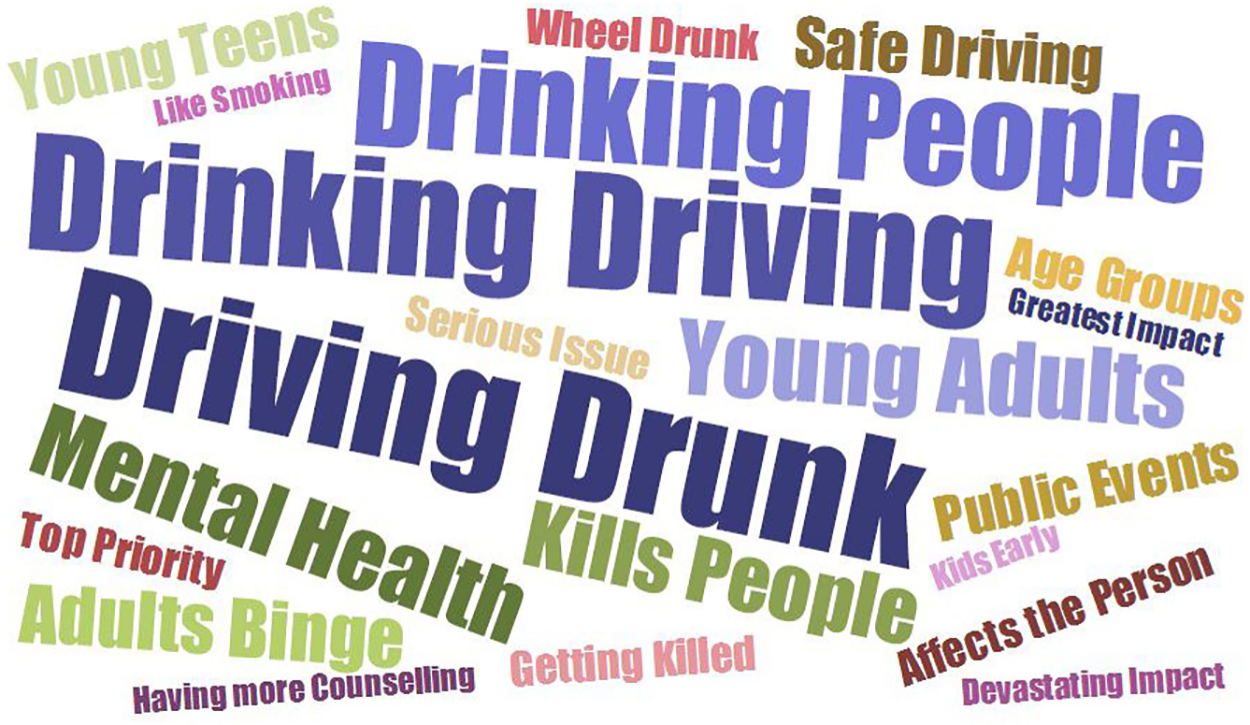
Word cloud representing the top alcohol concerns to work on first and why.

When asked what alcohol-related concerns to be worked on first, the themes of responses with highest frequency were (1) drunk driving; (2) access and availability of support services for the individual and family with alcohol addiction or mental illness; (3) underage drinking; (4) violence (including sexual violence) and public safety; (5) social norms of alcohol- frequently advertised, accessible; and (6) binge drinking. A word cloud was generated to represent the text responses, with the words that appeared most often in larger type (Fig 6).

## Discussion

This study used both quantitative and qualitative data to better describe the burden of alcohol consumption in the Ottawa population and is one of the first studies to highlight community concerns for alcohol-related social harms. Results showed that alcohol consumption is very common in Ottawa and patterns of consumption differ by sociodemographic and economic status. Moreover, contrary to the assumption that dependence upon alcohol account for greater burden of alcohol problems [29], our results suggested that the large proportion of low to moderate risk drinkers could have significant contribution to the burden. Similar findings have been described by Babor et al (2010) indicating that the large number of moderate-risk drinkers account for the greater proportion of alcohol-related harm and healthcare and community service costs rather than the relatively small number of high risk drinkers [30].

Patterns of consumption among men and young people aged 19 to 24 years place them at higher risk for the acute and chronic effects of alcohol. The observed gender and age differences in alcohol drinking behaviour and related harms are consistent with current literature indicating that men are more likely than women to not only consume alcohol, but consume it in larger quantities [9–11, 31]. Men who consume alcohol also experience more behavioural problems related to their drinking than women who consume alcohol [32, 33]. Furthermore, respondents of the Have Your Say online alcohol survey aged under 25 years cohort were least concerned about underage and binge drinking. This result is not surprising since binge drinking has been shown as the most common pattern of alcohol consumption among youth who drink alcohol [7, 34]. Although alcohol is the most commonly used drug among Canada’s youth [35], research has indicated that youth drink less often than adults, but when they drink, they tend to drink more (e.g. binge drinking) [36]. It is estimated that young people consume more than 90% of their alcohol by binge drinking [37]. The public attitudes towards binge drinking from our survey indicated that many young people feel binge drinking is not more risky than drinking smaller quantities and it is a matter of personal choice. Due to the difficulties in changing personal attitudes, intervention programs designed to change the way young people think about alcohol would be more effective in combination with other policy approaches [30].

Findings further indicated that alcohol contributes not only to health-related consequences such as injury, mental health conditions and chronic disease but that Ottawa residents are concerned for alcohol-related social problems for individuals, families and the community. Despite improvements on the incidence of drunk driving, drunk driving was the topic that respondents of the Have Your Say survey most consistently noted can harm the community and the innocent, non-drinker. They expressed concerns for alcohol-related domestic, sexual and other forms of violence (e.g. bar fights) and linked these to community concerns regarding public safety, to a need for better addictions services, and to underage drinking. However, written responses for those who were concerned about the issue of binge drinking described themed concerns that related to affects on the individual. Collectively, these results underscore the need to reframe social norms about alcohol use from solely a personal issue to a wider community issue and one with long-term health and social affects. A collaborative effort is necessary to encourage the population—particularly men and young adults—to adopt a culture of drinking in moderation in order to support lower risk alcohol use.

The estimated prevalence of alcohol consumption in Ottawa (83%) was higher than the estimated rate at the provincial level (78%), but comparable to the national (80%) level. The observed differences between Ottawa and the rest of the province may be explained by factors, such as: (1) *High income*. Ottawa is known to have the highest median income of large cities in Canada (median household total income of $79,634 before tax in Ottawa vs. $66,358 in Ontario) mainly because of the large number of public sector employees in the region—representing the highest paid as a group—and professional services firms’ employees [38]; (2) *Increased access and availability of alcohol*. The Ottawa population has increased access and availability of alcohol via its proximity with the province of Quebec, where alcohol is sold in grocery stores, convenience stores, and provincially-owned liquor stores for longer hours, at lower average prices (particularly for beer and liquor, which are favoured for binge drinking), and at a lower minimum age than in Ontario [39]; (3) *The higher proportion of immigrants in the province compared to Ottawa* (29% vs. 23%), mostly driven by Toronto and area’s large immigrant population [37]. Immigrants are known to drink less than non-immigrants in Canada [40]. This is supported by our findings indicating that immigrants were less likely than non-immigrants to report alcohol consumption in the past year, binge drinking, heavy drinking, and to exceed weekly limits.

Results also showed that people of high income are more likely to report alcohol consumption and binge drinking and to exceed weekly limits than those of low income. This supports, at least in part, current literature on the existing paradoxical relationship between alcohol consumption and SES, according to which people of high income have the same or higher level of alcohol consumption, but it is people of lower education and income who are at greater risk of alcohol-related morbidity and mortality [13–15]. While the mechanisms that explain such a relationship are not well understood, it was suggested that the differences in drinking behaviours (e.g. quality of the alcohol consumed) [41, 42], interact through clustering of risky lifestyle behaviours (e.g. heavy drinking and smoking) [43], and differential access to healthcare [44] may explain the association between risk of alcohol-attributable disease and SES. It also been suggested that social support and effect of neighbourhood deprivation on individual SES could explain this association [15, 45]. Future research is necessary to explore mechanisms explaining the relationship between alcohol consumption and SES in order to better inform the development of effective public health programs.

### Public health implications

Alcohol is the most commonly used drug in Canada. Our results indicate that its consumption and misuse have widespread impacts on individuals and their community, supporting the need for healthy public policy intended to encourage a culture of drinking in moderation. The seven internationally recognized alcohol policy areas that are evidence-informed and considered to be the most effective in the reduction of harm and health risks are: 1) regulation of the availability of alcohol, 2) alcohol taxation and price control, 3) marketing restrictions, 4) drink-driving countermeasures and prevention, 5) modification of the drinking context, 6) education and persuasion strategies and 7) access to treatment and early intervention services [29, 46, 47]. The current provincial political will favours increased alcohol access and availability, citing public support [16]; however, there are definite impacts of increased alcohol access and availability to increasing consumption rates on individual harms and second-hand effects on the community. This presents a challenge for municipal public health to continue to successfully educate the public and influence decision makers to adopt a health-lens in municipal alcohol policy approaches. Suggested municipal approaches include addressing alcohol pricing controls, ensuring early identification and support services are available for individuals or family members dealing with alcohol use disorders, strengthening zoning and licensing strategies to limit outlet density and hours of sales, and increasing regulation of public special-occasions events by developing alcohol management practices [26, 48].

Comprehensively addressing local alcohol-related harms goes beyond the mandate, capacity and resources of any one organization. To foster this support, the local health unit is proposing a community engagement approach with an aim to reframe alcohol use from an individual issue to a community. Stakeholders will be sought to help inform a local assessment of alcohol policies, which can support the community, raise awareness of harms, influence community social norms, and promote healthier communities [49].

### Limitations and strengths

This study has several strengths and limitations. First, with response rates in some cycles of the CCHS such as 66.8% in 2013, there is likely some non-response bias which could affect the validity of those estimates. However, Statistics Canada has adjusted the survey weights by redistributing weights of non-responding households or people to responding households or people with similar characteristics, such as geographic information, collection period, time and number of contact attempts in order to minimize potential bias associated with total non-response.

Second, the CCHS data used herein were not adjusted for the under-reporting of alcohol use. Indeed, self-reported alcohol consumption has been indicated to be significantly lower when compared to per capita alcohol sales [50]. Zhao et al. recently indicated that after adjusting for under-reporting of alcohol consumption, the percentage of drinkers aged 15 and over exceeding daily and weekly LRADG limits in Canada in 2008–2010 increased from 17% to 39% and 7 to 27%, respectively [51]. Future studies are needed to account for these self-reported biases in alcohol consumption. Given that the present study included individuals aged 19 years and over, it is possible that such underreporting is minimized. A multivariate analysis might show other relationships between the sociodemographic variables.

Third, paramedic data reported herein are likely underestimated because they include responses where the chief complaint or the paramedics’ assessment of the situation was alcohol intoxication or alcohol ingestion. It does not include the many other calls where alcohol was a factor, such as injuries due to falling while drunk. Furthermore, location is based on where the patient is assessed, which may or may not be the same as the patient’s place of residence. Not all patients assessed by a paramedic are transferred to an emergency department and a paramedic’s assessment may not align with the discharge diagnosis of an emergency department physician. The point density map does not take into account the population per square kilometre so the higher density in the downtown core may be due to the higher density of population in that area.

Fourth, alcohol-attributable ED visits do not capture ED visits that were partially attributable to alcohol such as those for injuries where alcohol is a contributory factor. Therefore, it underestimates the true burden of alcohol on ED visits.

Fifth, the AAH numbers presented here are only a portion of the far-reaching effects of alcohol in Ottawa. The effects are derived in part from self-reported alcohol consumption, which underestimates consumption. For 100% attributable conditions, all ages were used; however, for partially attributable conditions, only those aged 15 to 69 years could be included due to methodological limitations. As a result, the estimate of morbidity is likely higher [27, 52].

The sixth limitation is the lack of data on the drinking habits of those hospitalized or attending EDs. Such information would have allowed us to assess drinking patterns and risk behaviours associated with hospitalization and ED visits.

Lastly, the online alcohol survey used convenience sampling and did not collect gender, economic or education level to differentiate perceptions. Only those who understood French or English and had access to internet were able to participate in the survey.

Strengths of this study include the use of both quantitative and qualitative data to better understand the burden of alcohol consumption in the population, whereas previous studies examining the burden of alcohol consumption used one type of data. The present study included the broadest spectrum of alcohol-related health indicators, including prevalence and trend data, paramedic data, ED visits, hospitalization, and death. Assessing a broad spectrum of health indicators is important because it provides a more complete picture of the burden of alcohol on the population. Finally, our study used qualitative data as an important tool to capture social or second-hand effects of alcohol consumption on individuals, families and the community, which can be difficult to capture with quantitative data.

### Conclusions

Alcohol has far-reaching short-term, long-term and second-hand effects on health and safety; Ottawa’s high rates of drinking put the population at higher risk for these effects. Our results highlight the need for healthy public policy intended to encourage a culture of drinking in moderation in Ottawa to support lower risk alcohol use, particularly among men and young adults, rather than increased access. Advancing effective alcohol policies is a way to promote moderate alcohol consumption and create healthier communities. Although policy interventions can be implemented in isolation of each other, multiple, coordinated interventions are more effective. A collaborative effort is necessary to encourage the population to adopt a culture of drinking in moderation in order to support lower risk alcohol use.

## Supporting information

S1 Table International Classification of Disease (ICD-10) codes used to classify alcohol-attributable ED visits, hospitalizations and deaths. (DOCX)
