# Supplementary material for: The burden of alcohol-related morbidity and mortality in Ottawa, Canada
Source: PLoS One. 2017 Sep 28;12(9):e0185457. doi: 10.1371/journal.pone.0185457 (PMC5619783; doi:10.1371/journal.pone.0185457)
Supplement: S1 Table — (DOCX) [file pone.0185457.s001.docx]

S1 Table. International Classification of Disease (ICD-10) codes used to classify alcohol-attributable ED visits, hospitalizations and deaths

| **Description** | **ICD-10 codes** | **Alcohol-attribution** |
| --- | --- | --- |
| Mental health conditions | F10.0 – F10.9, G31.2, G62.1 | 100% |
| Chronic disease | I42.6, K29.2, K70, K86.0 | 100% |
| Alcohol poisoning | X45, X65, Y15 | 100% |
| Fetal alcohol spectrum disorder | Q86.0 | 100% |
| Injuries | X60 – X64, X66 – Y09, Y87.0, Y87.1, Y35; X10 – X44, X46 – X59, W00 – W19, X00 – X09, W65 – W74, W20 – W64, W75 – W99, Y85 – Y86 | Partial |
| Digestive diseases | K74, K80, K85, K86.1 | Partial |
| Cancer | C00 – C15, C22, C32, C50, D00 – D48 |  |
| Low birth weight | P05 – P07 | Partial |
| Cardiovascular disease | I10 – I15, I20 – I25, I47 – I52, I60 – I69, I85, I97.0, I97.1, I98.1 | Partial |
| Depression | F32 – F33 | Partial |
| Epilepsy | G40 – G41 | Partial |
| MVTC |  | Partial |
